# Size-exclusion chromatography combined with DIA-MS enables deep proteome profiling of extracellular vesicles from melanoma plasma and serum

**DOI:** 10.1007/s00018-024-05137-y

**Published:** 2024-02-14

**Authors:** Evelyn Lattmann, Luca Räss, Marco Tognetti, Julia M. Martínez Gómez, Valérie Lapaire, Roland Bruderer, Lukas Reiter, Yuehan Feng, Lars M. Steinmetz, Mitchell P. Levesque

**Affiliations:** 1 Department of Dermatology, University Hospital Zurich, University of Zurich, Schlieren, Switzerland; 2 Biognosys AG, Schlieren, Switzerland; 3 Stanford Genome Technology Center, Stanford University, Palo Alto, CA USA; 4 Department of Genetics, Stanford University School of Medicine, Stanford, CA USA; 5 Genome Biology Unit, European Molecular Biology Laboratory, Heidelberg, Germany

**Keywords:** Extracellular vesicle, Exosome, Melanoma, Proteomics, Mass spectrometry, Size-exclusion chromatography

## Abstract

Extracellular vesicles (EVs) are important players in melanoma progression, but their use as clinical biomarkers has been limited by the difficulty of profiling blood-derived EV proteins with high depth of coverage, the requirement for large input amounts, and complex protocols. Here, we provide a streamlined and reproducible experimental workflow to identify plasma- and serum- derived EV proteins of healthy donors and melanoma patients using minimal amounts of sample input. SEC–DIA-MS couples size-exclusion chromatography to EV concentration and deep-proteomic profiling using data-independent acquisition. From as little as 200 µL of plasma per patient in a cohort of three healthy donors and six melanoma patients, we identified and quantified 2896 EV-associated proteins, achieving a 3.5-fold increase in depth compared to previously published melanoma studies. To compare the EV-proteome to unenriched blood, we employed an automated workflow to deplete the 14 most abundant proteins from plasma and serum and thereby approximately doubled protein group identifications versus native blood. The EV proteome diverged from corresponding unenriched plasma and serum, and unlike the latter, separated healthy donor and melanoma patient samples. Furthermore, known melanoma markers, such as MCAM, TNC, and TGFBI, were upregulated in melanoma EVs but not in depleted melanoma plasma, highlighting the specific information contained in EVs. Overall, EVs were significantly enriched in intact membrane proteins and proteins related to SNARE protein interactions and T-cell biology. Taken together, we demonstrated the increased sensitivity of an EV-based proteomic workflow that can be easily applied to larger melanoma cohorts and other indications.

**Supplementary Information:**

The online version contains supplementary material available at 10.1007/s00018-024-05137-y.

## Introduction

Extracellular vesicles (EVs) are released by almost all body cells, including tumor cells, and are important mediators of metastasis formation by regulating cellular homeostasis and intercellular communication [1–3]. As EVs traffic through body fluids, they can be recovered from liquid biopsies and hold great promise for non-invasive biomarker discovery and disease detection [4–7]. Exosomes are the most prominent subcategory of EVs. They are 50–200 nm-sized, lipid-bilayer enclosed particles, which are formed within the endocytic pathway by inward budding of multivesicular bodies (MVBs) and subsequent release of the intraluminal vesicles through fusion of MVBs with the plasma membrane [8]. Tetraspanins CD9, CD63, CD81, and endosomal sorting complexes required for transport (ESCRT) protein TSG101 and ESCRT associated protein Alix are classically used as exosome markers, although recent evidence suggests that these markers are heterogeneously expressed on EVs [9–14]. We therefore refer to 50–200 nm-sized vesicles collectively as EVs. Major EV functions include sequestering and trafficking of bioactive molecules, such as proteins, small RNAs, mRNAs, lipids, and metabolites [10, 15–19]. Moreover, surface receptors can mediate contact of EVs with specific target cells and induce EV uptake via receptor-mediated endocytosis or receptor activation and downstream signalling [3, 20–22]. Notably, differential integrin composition on the surface of EVs has been attributed to direct organotropism of breast cancer, exosomal integrins α_6_β_4_ and α_6_β_1_ associating with lung metastasis, and exosomal integrin α_v_β_5_ with liver metastasis, respectively [23].

Recent evidence suggests that EVs play a key role in melanoma, the deadliest form of skin cancer [24]. Melanoma arises by malignant transformation of the pigmented cells in the skin, the melanocytes, and is characterized by high metastatic potential [25]. EVs play a role in the metastatic process at several stages including reprogramming of cancer associated fibroblast and stimulating angiogenesis and lymphangiogenesis [26–31]. For example, secretion of NGFR by melanoma-derived EVs was suggested to induce lymphangiogenesis in lymphatic endothelial cells (LECs) [32]. Melanoma exosomes also stimulate vascular leakiness by reprogramming bone marrow progenitor cells via the receptor tyrosine kinase MET [31]. Moreover, EVs regulate melanoma communication with the immune system and melanoma-derived EVs express PD-L1 on their surface and aid tumour immune surveillance evasion [33, 34]. Thus, exosomal PD-L1 has been proposed as a predictor of treatment response and could be used for real-time monitoring of melanoma disease progression [35].

Despite the significance of EV proteins, unbiased proteomics analysis of melanoma EVs isolated from blood liquid biopsies remains difficult. To date, only a handful of pioneer studies utilized mass spectrometry-based workflows to analyze blood-derived EVs from melanoma patients with modest depth of coverage [36–39]. Limited application and reproducibility of EV biomarker discovery is attributable to both biological and technical factors. On the one hand, large inter- and intra-patient variability challenges identification of reliable biomarkers. On the other hand, the presence of high-abundant proteins in EV preparations makes it difficult to detect biomarkers present in lower abundance. Accumulation of high abundance proteins in EV preparations results either from improper separation during the EV isolation process or the natural coating of blood EVs with a blood–protein corona [40, 41]. Some protein contaminants can be removed depending on the choice of EV isolation method; however, removal of the protein corona remains a barrier to comprehensive proteome profiling.

Here, we couple size-exclusion chromatography (SEC), which separates EVs based on size, to data-independent acquisition (DIA) mass spectrometry. SEC–DIA-MS showed high technical reproducibility and improved quantitative performance compared to previous melanoma studies. By applying SEC–DIA-MS to plasma and serum of melanoma patients in parallel with quantitative proteome profiling of depleted and native plasma and serum, we show that blood-derived EV-protein pools are distinct from unenriched blood protein pools. Moreover, plasma- and serum-derived EVs display a more distinct protein signature in melanoma patients vs healthy control subjects compared to native and depleted plasma or serum, albeit in a small test cohort. Therefore, applying SEC–DIA-MS to a larger cohort will improve the sensitivity and robustness of biomarker discovery and disease detection in melanoma.

## Materials and methods

### Plasma/serum sample collection and pre-processing

Healthy donor serum and plasma samples were obtained from the blood donation center (Blutausgabe, CH-8952 Schlieren) under the ethical approval BASEC.Nr.2018-00194. Melanoma patient’s serum and plasma samples were obtained from the melanoma biobank of the University Hospital Zurich under the ethical approval BASEC.Nr.2017-00494. The samples were age- and gender-matched. Serum samples were collected in orange-capped tubes (BD Vacutainer CAT Plus, cat. 367896) and plasma samples in purple-capped EDTA tubes (BD Vacutainer K2 EDTA Plus, cat. 367525). Samples were immediately centrifuged at 1600 g for 10 min. The supernatant was collected without touching the buffy coat layer and centrifuged at 3000 g for 10 min. The supernatant was transferred to a cryo-tube and stored at −80 °C. The freeze cycle was limited to one prior to EV isolation. Before EV isolation, 220 µL of serum or plasma were pre-processed by centrifugation at 1500 g for 10 min (4 °C), and at 10,000 g for 30 min (4 °C). For each centrifugation step, the pellet was discarded, and the supernatant collected for further processing.

### Plasma and serum pre-processing and EV isolation

A total of 200 µL of supernatant was used as input for SEC. SEC was done with qEV original (legacy) columns with a pore size of 70 nm (IZON, SP1) as previously described [42]. Dulbecco's phosphate-buffered saline (DPBS) (Gibco, 14190250) was used as solvent for SEC fractionation. Prior to EV isolation, DPBS was filtered through a vacuum filtration unit containing a PES membrane filter with a pore size of 0.2 µm (TPP, #99500) and degassed for at least 1 h to remove dissolved gases. Prior to SEC fractionation, the column was washed with at least 20 mL of filtered and degassed DPBS. A constant solvent flow was maintained to avoid drying of the columns. 3 mL (Fraction 1–6) were kept as void for quality control analysis and 2 mL of EV-enriched suspensions (fraction 7–10) were collected for downstream processing. qEV columns were used maximally five times. To determine the contamination with free protein, the 280 nm absorption of 1 µL of void fractions 1–6, EV-containing fractions 7–10, and protein-enriched fractions 11, 12, 13–22 and 23–32 was measured spectrophotometrically using NanoDrop ND-1000 (Thermofischer, USA).

### Concentration of EVs for proteomic analysis

100 µL of concentration beads (qEV Concentration Kit, IZON) were added to 2 mL of fraction 7–10 and incubated on a rotating wheel for 1 h at 22 °C. The bead suspension was centrifuged at 16,800 g for 10 min at 22 °C and the supernatant pipetted off without touching the bead pellet. The Eppendorf tube with the bead pellet was snap frozen and stored at − 80 °C before processing for mass spectrometry.

### Reproducibility analysis

To assess the technical variability of the workflow, plasma and serum samples were pooled at equal amounts. Subsequently, 200 µL of the pooled plasma and serum, respectively, were used as input for SEC of each of the technical triplicates. Further processing of the triplicates was identical to the study samples.

### Plasma and serum depletion

Plasma and serum depletion was performed using the multi affinity removal column human-14, 4.6 × 50 mm^2^ (Agilent Technologies) as previously described by Tognetti et al. [43].

### Protein aggregation capture digestion

EV bead pellets were resuspended in 100 µL of lysis buffer (300 mM Tris, 5% SDS, pH6) and denatured at 95 °C for 5 min. Native plasma and serum samples were prepared with the same procedure. Sample processing batches were block-randomized by sample type and subsequently processed with a protein aggregation capture (PAC) protocol, as described elsewhere [43, 44].

### Peptide cleanup

Acidified peptides were purified by solid-phase extraction (SPE) with an HLB 96-well µElution plate (Waters) according to the manufacturer’s instructions. Subsequently, resuspended in 0.1% formic acid spiked with iRT kit (Biognosys). For native and depleted samples, peptide concentrations were assessed with Micro BCA Protein Assay Kit (Thermo Fisher Scientific) according to the manufacturer’s instructions.

### High pH reversed phase (HPRP) fractionation for library generation

The purified peptide mixtures were further fractionated using HPRP as previously described by Tognetti et al. [43]. Briefly, either 80 µg of EV or 200 µg of depleted or native peptides were pH-adjusted and afterwards fractionated to a total of 12 peptide pools. The pools were dried and subsequently processed as described above to prepare them for LC–MS/MS acquisition.

### Mass spectrometric acquisition

For DIA LC–MS/MS measurements, 3.5 µg of for native and depleted or 11 µL of EVs of peptides per sample were injected on an in-house packed reversed phase C18 column (75 µm*60 cm) on a Thermo Scientific™ NeoVanquish UHPLC nano-liquid chromatography system connected to a Thermo Scientific™ Orbitrap™ Exploris 480™ mass spectrometer equipped with a Nanospray Flex™ ion source and an FAIMS Pro™ ion mobility device (Thermo Scientific™). LC solvents were A: water with 0.1% FA; B: 80% acetonitrile, 0.1% FA in water. The nonlinear LC gradient was 1–50% solvent B in 172 min followed by a column washing step in 90% B for 5 min, and a final equilibration step of 1% B for 1 column volume at 64 °C with a flow rate set to a ramp between 500 and 250 nl/min (min 0: 500 nL/min, min 172: 250 nL/min, washing at 500 nL/min). The FAIMS-DIA method consisted per applied compensation voltage of one full- range MS1 scan at 120,000 resolution from 350 to 1650 m/z and 34 DIA segments at 30,000 resolution as adopted from Bruderer et al. [87] and Tognetti et al. [89]. For DDA acquisition, the same gradient and m/z range were used as for the DIA and the cycle time was fixed per CV. The MS data, the spectral libraries, and the quantitative data tables have been deposited to the ProteomeXchange Consortium via the MassIVE [45] repository with the data set identifier MSV000090893. The saved projects from Spectronaut can be viewed with the Spectronaut Viewer (www. biognosys.com/spectronaut-viewer).

### Mass spectrometric data analysis

The collected RAW files from DIA runs were analyzed batch-wise for serum and plasma using Spectronaut (Biognosys, Version18.1.230626.50606) with the directDIA™ workflow using additional RAW files from DDA runs of HPRP-fractionated samples to create a hybrid library. In brief, false discovery rate (FDR) was set at 1% at the PSM, peptide and protein group level with a maximal number of two missed cleavages, as well as variable (N-terminal acetylation and methionine oxidation) and fixed (cysteine carbamidomethylation) modifications. Deviations from standard workflow settings included a run-wise protein q-value cutoff of 0.01 and no normalization was applied among runs, to preserve the inherent biological variability within EV samples (Supplementary Figure S1a, b). FASTA files were generated from UniProt sequences (Homo Sapiens, 2023-07-01, 20423 entries) database.

### Data analysis

Sample quality of plasma samples was assessed by determining the platelet, erythrocyte, and coagulation scores. Here, the ratio of summed intensities of all proteins to the intensity sum of a selected protein panel was calculated, as previously described by Geyer et al. [46]. Similarly, a score to assess the depletion efficiency using similar calculations but the depleted proteins as panel was implemented. Hierarchical clustering was performed on the complete protein profiles shared between healthy and melanoma samples using the R-package “complexHeatmap” with standardized protein quantities (*z*-scores). Euclidian distances were calculated, and samples clustered using a complete linkage algorithm. Principal component analysis (PCA) was performed on the complete protein profiles shared between healthy and melanoma samples using R-package “prcomp”. Membrane protein coverage analysis was performed using UniProt sequences (Homo Sapiens, 2022-01-01, 20375 entries) database annotated with topology information. Identified peptides were subsequently categorized into intracellular, extracellular, or mixed based on annotation data. Peptide-to-protein structure mapping was done by annotating the identified stripped peptide sequences onto AlphaFold’s [47] protein structure database along with model confidence information. Topological domain information was retrieved from UniProt sequence database, as described above. The clusterProfiler package was used for enrichment analysis of differentially expressed gene sets by GO terms and KEGG pathways. The GO term analysis was restricted for cellular components onotology with gene set sizes between 50 and 5000 genes, while for KEGG pathways, standard settings were used. To generate a protein–protein interaction network, the identified proteins of the KEGG pathways associated with T-cell biology were analyzed with the string database (https://string-db.org/) and visualized with Cytoscape (V3.9.1).

### Nano-flow cytometry measurements and analysis of EVs

To quantify EV particle number and size distributions, NanoFCM measurements were performed according to Tian et al. [48]. Briefly, 100 µL of EV suspensions were used as input for nano-flow cytometry (NanoFCM, Inc., Xiamen, China). Prior to measurements, lasers were calibrated with quality control beads (Quality Control Nanospheres, Nano FCM; 250 ± 5 nm) that were diluted 1:100 in filtered DPBS (Total volume 100 µL). For size calibration, monodisperse silica nanospheres (cat. S16M-Exo, NanoFCM; 68–155 nm) were diluted 1:100 in filtered DPBS (total volume 100 µL) and measured for 1 min on the NanoFCM. For each size standard and sample measurement, the suspension was boosted for 45 s prior to acquisition to ensure proper pressurization and acquired for 1 min. Samples were diluted, such that total events were in the range of 2000–12000 to minimize swarm detection effects. Between each measurement, the inlet was washed with 100 µL cleaning solution. After a 45 s boost, the cleaning solution was removed and the inlet was briefly rinsed in 100 µL filtered water. Data analysis and export were done using NanoFCM software (NF Profession Version 1.15). Conversion of flow rates and side scatter intensities to vesicle concentrations and sizes was done with the calibration curves of the measured quality control beads and size standard nanospheres, respectively.

### Transmission electron microscopy

500 µL of plasma and serum of healthy donors were used as input for SEC. 2 × 500 µL of fraction 7–10 were loaded onto a PES filter (Vivaspin® 500, cat. VS0151, Sartorius) with a molecular weight cut-off (MWC) of 300 kDa and centrifuged for 10 min at 2000 g, 12 °C (each round) to concentrate the EV preparations prior to TEM processing. For the negative staining a carbon-coated 300 mesh nickel grid was clamped with an inverted tweezer. A 5 µL drop of sample solution was added onto the grid. After 1 min incubation, the sample solution was blotted away with a piece of filter paper (Whatman hardened ashless) and a 5 µL drop of 1% Uranyl acetate water was immediately added. The grid was stained for 1 min and the uranyl acetate solution blotted away. Finally, the grid was air-dried in the tweezers and placed in a grid box. Sample were imaged in a Talos 120 transmission electron microscope at 120 kV acceleration voltage equipped with a bottom mounted Ceta camera using the Maps software (Thermo Fisher Scientific, Eindhoven, The Netherlands).

## Results

### Generation of a protein atlas of different blood compartments (native, depleted, and EVs) of plasma and serum

**Fig. 1:**
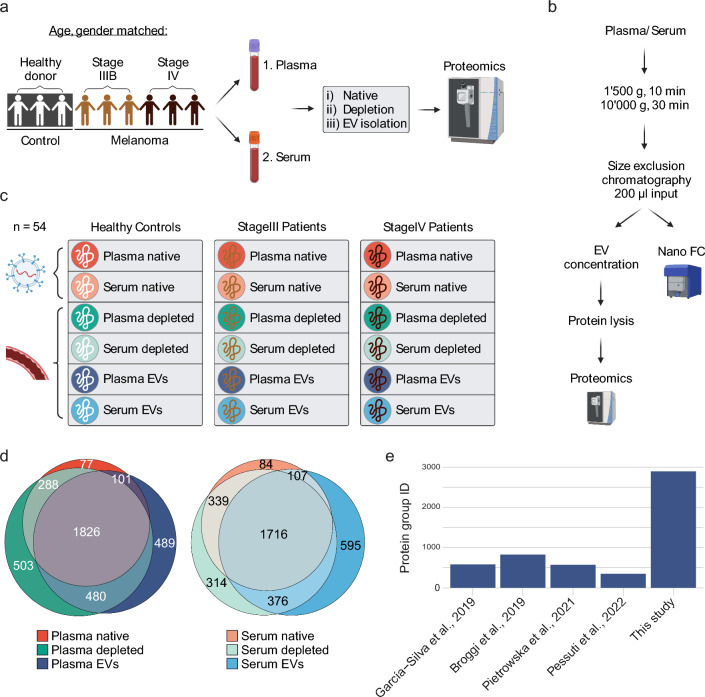
Study workflow and overall protein identification. **a** Study outline. Plasma and serum samples were collected from a cohort composed of age- and gender-matched healthy donors (*n* = 3), stage III (*n* = 3), and stage IV (*n* = 3) melanoma patients yielding 18 samples in total. For all 18 samples, three different blood compartments (native, depleted, and EVs) of plasma and serum were analyzed by mass spectrometry yielding 54 samples in total. **b** EV isolation workflow from 200 µL of plasma or serum by size exclusion chromatography and subsequent quality control by nano-flow cytometry (Nano FC). **c** Generation of a protein atlas for different blood compartments. Protein groups were determined in three different blood compartments (native, depleted, and EVs) of plasma and serum, respectively. For each plasma and serum compartment proteins from healthy donor, stage III and stage IV melanoma patients were quantified. **d** Venn diagram of total protein group identifications of blood compartments across all analyzed patients. Note that the protein group identifications of native, depleted, and EV compartment show similar overlaps in plasma and serum samples. **e** Number of protein groups identified in plasma-derived EVs in previous melanoma studies and our study

Liquid blood biopsies provide non-invasive means to discover biomarkers for melanoma diagnosis, treatment decisions, monitoring, and prediction of overall survival by providing insights into the disease state of patients. Nevertheless, the high dynamic range of protein abundance in blood and blood-derived EV preparations impede biomarker discovery. To overcome this challenge, we developed SEC–DIA-MS, an integrated workflow combining size-exclusion chromatography, EV concentration, and DIA-mass spectrometry that enables deep profiling of the proteome content of enriched EVs. To assess whether our method has the potential to leverage melanoma biomarker discovery, we analyzed matching plasma and serum samples from a cohort of gender- and age-matched healthy donor (*n* = 3), stage III (*n* = 3), and stage IV (*n* = 3) melanoma patients (Fig. 1a). Briefly, we profiled the proteome of nine plasma and nine corresponding serum samples: (i) without processing (native), ii) with high-abundance blood protein depletion, and iii) with EV enrichment. EVs were isolated by size-exclusion chromatography (SEC) from 200 µL of plasma or serum input, respectively. EV-containing fractions were concentrated on affinity beads before protein lysis and mass spectrometry acquisition (Fig. 1b). Our integrative approach yielded a comprehensive protein atlas of native, depleted, and EV blood (plasma and serum) compartments of healthy control, stage III, and stage IV melanoma patients (Fig. 1c). Overall, more proteins were shared than unique and protein overlap was similar across the three preparations for both plasma and serum (Fig. 1d). For example, 1826 (48.5%) and 1716 (48.6%) of identified proteins were shared across all compartments in plasma and serum samples, respectively. Percentages of unique proteins in a compartment ranged from 2.0 ± 0.4% in native to 13.0 ± 2.8% in EV samples up to 13.4 ± 4.5% in depleted blood. In total, we detected 2896 protein groups in plasma-derived EVs. This is 3.5–8.4 × higher than protein groups identified in the previous studies that performed proteomic profiling of plasma-derived EVs in melanoma patients (Fig. 1e) [36–39]. Taken together, our approach allowed for significantly improved protein detection and thus paves the way for biomarker discovery from (EV-enriched) blood biopsies. Moreover, the minimal amount of required input allows for the preservation of precious patient material.

### Plasma- and serum-derived EVs isolated by SEC are consistent with size, morphology, and protein composition of exosomes

**Fig. 2:**
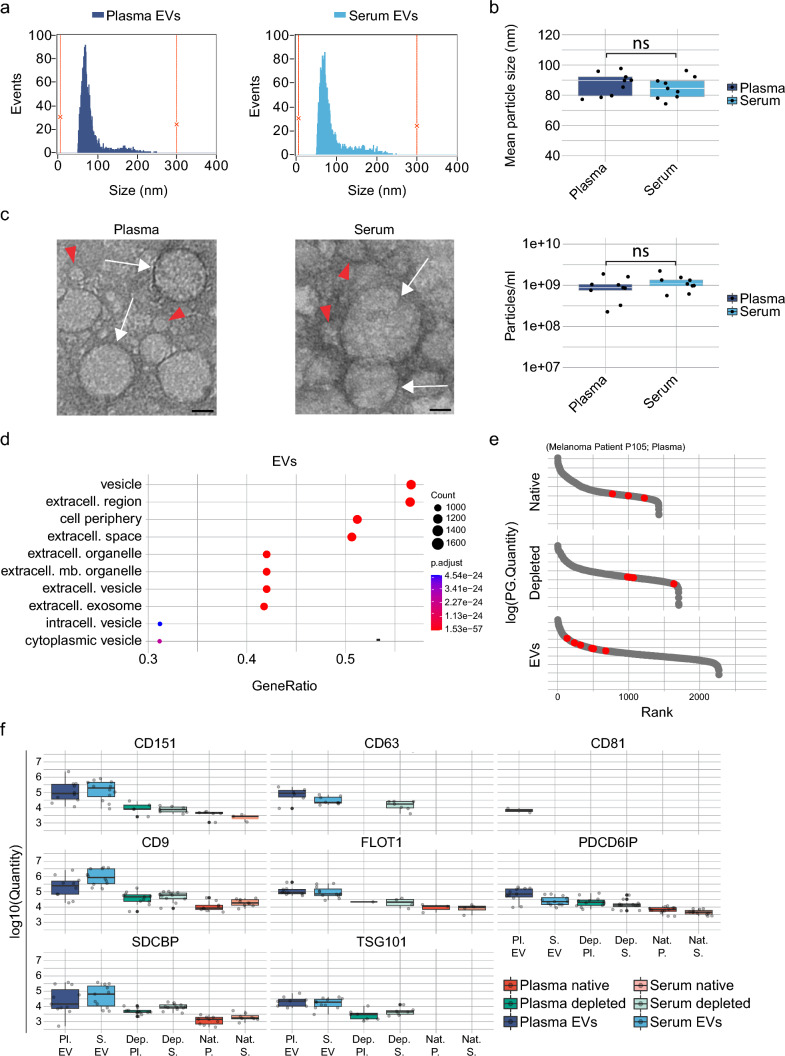
Characterization of EVs isolated from plasma and serum of healthy donors and melanoma patients**. a** Size profile of a representative plasma- and serum-derived EV sample isolated by size-exclusion chromatography and acquired by nano-flow cytometry. **b** Mean particle size (top) and particle number (bottom) of EVs isolated from 200 µL of plasma and serum, respectively. Statistical significance was determined with a two-tailed t test for independent samples, ns stands for not significant using a p-value significance cutoff of 0.05. **c** High-resolution transmission electron microscopy of plasma and serum EVs. White arrows depict EVs and red arrowheads potential lipoproteins/protein aggregates. The scale bar is 20 µm. **d** GO terms in the enrichment analysis of proteins identified in plasma and serum EVs against all identified proteins. **e** Average protein intensity plotted vs protein rank of eight exosome markers (red dots). **f** Quantification of exosomal markers including tetraspanins CD151, CD63, CD81, CD9, scaffolding protein flotillin FLOT1, luminal vesicle proteins Alix (PDCD6IP) and TSG101, and putative universal exosome marker Syntenin-1 (SDCBP) in plasma- and serum-derived EVs. Black lines represent the median and the upper and lower limit of the box depicts the 25 and the 75 percentile, respectively

To characterize EV populations, we measured the particle concentration and size of all 18 EV preparations by nano-flow cytometry (Fig. 2a, b, Supplementary Fig. S2). The particle size distributions ranged from approximately 50 nm to 260 nm in plasma and from 50 to 270 nm in serum samples, consistent with the presence of exosomes (Fig. 2a, Supplementary Fig. S2a). The mean size of plasma- and serum-derived particles was on average 86.8 nm and 85.9 nm, respectively, revealing no significant particle-size difference between these sample types (Fig. 2b, top, Supplementary Fig. S2a). Similarly, particle concentrations of plasma- and serum-derived EVs were comparable, yielding an average of 9.53E + 08 or 1.18E + 09 particles from 200 µL of plasma or serum input, respectively (Fig. 2b, bottom). The particle number correlated well with the number of protein identifications in plasma samples (Supplementary Fig. S2b). To assess contamination with free proteins in the EV preparations, we measured absorption at 280 nm across different fractions of the SEC. Absorptions of fractions (1–6), which correspond to the void volume, and the subsequent EV-containing fractions (7–10) showed efficient exclusion of free proteins in plasma and serum samples (Supplementary Fig. S2c). Transmission electron microscopy showed intact vesicles within the expected size range (Fig. 2c). To confirm the presence of exosomes at the protein level, we performed a combined GO term analysis of all blood EV samples, which showed a significant enrichment of GO terms associated with vesicles (*p*-adjusted = 1.54E − 57) and extracellular exosomes (*p*-adjusted = 3.41E − 55) (Fig. 2d). In addition, comparison to the top 100 proteins of the Exocarta database [49] showed a 92% overlap. Next, we determined the abundance of classical exosome markers in plasma- and serum-derived EVs in comparison to native and depleted plasma and serum samples. Assessed proteins, including tetraspanins CD151, CD63, CD81, CD9, ESCRT protein TSG101, ESCRT-associated protein Alix (encoded by the PDCD6IP gene), Flotillin (FLOT1), and exosome marker syntenin-1 (SDCBP), were enriched in EVs compared to native and depleted samples (Fig. 2e, f, Supplementary Fig. 3a). It is noteworthy that CD81 was exclusively detected in plasma samples and not in any other sample type. This observation suggests that CD81 may either be less abundant than other EV markers in these sample types or more difficult to detect by mass spectrometry (Fig. 2f, Supplementary Fig. 3b, c). In contrast, nuclear markers (e.g., ASH2L, PAF49, CENPA, and CHOP), apoptotic protein BCL2, ER marker ATP2A2, and Golgi markers COG2, GOLGA2 were absent in all EV populations (Supplementary Fig. S4a) and other organelle markers were mostly reduced or unchanged compared to depleted plasma and serum (Supplementary Fig. S4b). In summary, plasma and serum EVs isolated with SEC were consistent with size, morphology, and protein composition of exosomes.

### Quality control of SEC–DIA-MS and high-abundant blood protein depletion

Reproducibility of the SEC–DIA-MS workflow was done on three technical replicates of a pooled sample comprising all biological replicates of the respective compartment (native, depleted, and EVs). Particle concentration, size, and protein contamination of the EV-containing fractions (fractions 7–10) were consistent among the replicates (Supplementary Fig. S5a–d), showing reproducible EV separation. As observed for individual samples, particle size and number did not differ significantly between plasma- and serum-derived EVs (Supplementary Fig. S5b–d). Overall, the same proteins were identified (Supplementary Fig. S5f) and the coefficient of variation across the entire workflow was low and comparable between plasma and serum samples with median values of 14.4.%/23.8% in native plasma/serum, to 18.9%/19.5% in depleted plasma/serum, and to 37.7%/38.9% in plasma/serum EVs (Supplementary Fig. S5e, g). Expectedly, native samples showed the least depth in protein, peptide, and precursor identifications, indicating that proteomic profiling of depleted blood and SEC–DIA-MS are superior (Supplementary Fig. S5g). Thus, combined with the good reproducibility, those approaches are preferred over profiling native blood. Next, we assessed the efficiency of the high-abundance blood protein depletion. In our workflow, plasma and serum depletion was achieved using antibodies against high-abundance proteins A2M, ALB, APOA1, APOA2, C3, FGA, HP, IGHA1, IGHM, ORM1, SERPINA1, TF, and TTR. The depletion score [46] of depleted samples was on average approximately 1.5 and three times higher compared to EVs and native samples, and except for A2M, APOA2, IGHM, and FGA, all targeted proteins were largely diminished in depleted blood. Specifically, the abundance of albumin (log_2_fc = 8.1 for native vs. depleted) and ORM1 were strongly reduced (log_2_FC = 8.7 native vs. depleted) confirming that high abundant proteins are efficiently removed from plasma and serum (Supplementary Fig. S6a,b). Notably, blood-derived EVs showed a higher depletion score than native blood samples and a pronounced reduction of several high abundant proteins with APOA2, FGA, and IGHM as exceptions (Supplementary Fig. S6a, b). Platelet protein, erythrocyte-associated proteins and coagulation proteins were diminished in EVs compared to depleted blood, although not down to the level of native samples (Supplementary Fig. S6c–h). In addition, melanosome proteins were abundant in EV samples (Supplementary Fig. S6i). Notably, the melanosome protein Rab38 was exclusively present in melanoma EVs while absent in healthy donor EVs (Supplementary Fig. S8b). Overall, the workflow resulted in high-quality proteomic profiling of either the free circulating or EV-embedded plasma/serum proteome.

### Plasma and serum EV proteomes diverge from the corresponding unenriched sources and display larger changes between healthy donors and melanoma patients

**Fig. 3:**
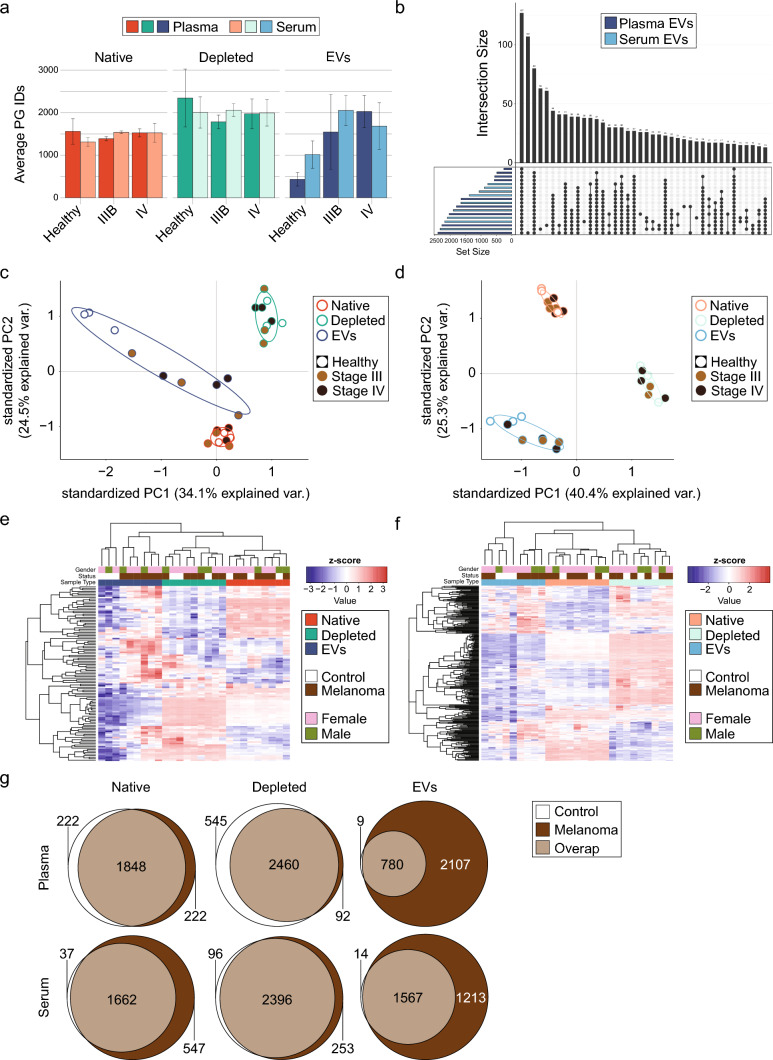
Protein depletion and EV isolation improve protein identifications compared to untreated (native) plasma or serum. **a** Protein groups identified in plasma and serum blood compartments across healthy control, stage III and stage IV patients. **b** UpSet plot showing the number of shared proteins among all analyzed EV-derived plasma and serum samples, respectively. The horizontal bars (blue) show the number of proteins identified in each individual sample, while the vertical bars (black) represent the number of overlapping proteins per subsets, which are visualized by the dotted lines. **c**, **d** Principal component analysis based on complete protein group identifications shared across all analyzed plasma (**c**) and serum (**d**) samples, respectively. The first two dimensions separate data points into native, depleted, and EV-derived clusters with PC1 explaining 34.1% and 40.4% of the variances for plasma and 24.5% and 25.3% for serum samples. 115 proteins were used for plasma samples and 299 proteins for serum samples. The corresponding ellipses represent sample concentration for native (red), depleted (green) and EV-derived (blue) samples around the mean. Note that among EV-derived samples, specimens from healthy control patients cluster apart. **e**, **f** Hierarchical clustering of Z-scored protein quantities for shared proteins of plasma (**e**, *n* = 115) and serum (**f**, *n* = 299) samples. Native blood samples are overlayed in red, depleted blood samples in green and EV-derived blood samples in blue. Above, samples from healthy controls are overlayed in white and samples from melanoma patients are overlayed in brown. On top, gender categorizations are overlayed, annotating samples derived from female patients in pink and samples derived from male patients in olive. **g** Venn diagram displaying the overlap of healthy control and melanoma protein identifications among native (left), depleted (middle), and EV-derived (right) plasma (top) and serum (bottom) biopsies

In line with the reproducibility analysis, we observed a 1.4-fold reduction in protein group identifications in native compared to depleted blood biopsies in all individual samples (Fig. 3a). In addition, there was a trend towards fewer protein identifications in EVs derived from healthy donor plasma and serum biopsies in comparison to melanoma patient biopsies (Fig. 3a). In all three blood compartments, there was a high overlap of protein identifications across samples, whereas EV-derived samples showed larger differences across samples compared to native and depleted blood samples (Fig. 3b, Supplementary Fig. S7a, b), likely due to biological variability among human subjects. The diversity of protein quantities across different blood compartments was evaluated with principal component analysis (PCA). The first principal component (PC1) and the second (PC2) separated EV-derived, depleted, and native blood compartments in three discrete clusters for both sample types: plasma, and serum (Fig. 3c, d). PC1 accounted for 34.1% and 40.4% and PC2 for 24.5% and 25.3% of the variability in plasma and serum samples, respectively. This implied the enrichment of distinct protein populations in EV-derived, depleted, and native blood compartments. Consistently, hierarchical clustering of plasma (Fig. 3e) recapitulated the dissimilar nature of EV-derived, depleted, and native plasma proteomes, whereas gender and age had no confounding effect. Similarly, native samples formed a distinct cluster in the heatmap of serum samples, but general differences between EV and depleted serum were less pronounced (Fig. 3f). Despite the modest patient number (*n* = 9), healthy controls separated from melanoma patients in the EV-derived plasma proteome (Fig. 3e) and formed a separate cluster. However, no clear distinctions were revealed between stage III and stage IV melanoma patients, likely due to the small cohort size. Based on the uniqueness of the EV proteome and the clear separation of plasma EVs from healthy donors and melanoma patients, we further evaluated the ability of EV protein profiles to identify potential melanoma biomarkers. For this purpose, we first compared protein identifications between healthy controls and melanoma patients in the six different compartments (Fig. 3g). In both native and depleted plasma and serum, only a few proteins were unique to the disease status (222 and 547 in native and 92 and 253 in depleted plasma and serum, respectively). In contrast, as we expect melanoma cells to secrete more diverse EVs than healthy individuals, EV samples showed the largest percentages of proteins specifically present in melanoma patients: 2107 proteins (73.0%) and 1213 proteins (43.6%) were exclusively detectable in melanoma plasma- and serum-derived EVs, respectively. Analogously, differential abundance analysis revealed up to 348 (plasma, serum: 257) proteins upregulated in melanoma EVs, whereas native and depleted blood showed fewer upregulated proteins in melanoma patients (38 in depleted plasma, 59 in depleted serum, 30 in native plasma, and 48 in native serum) (Supplementary Fig. S8a). The single upregulated protein in native serum overlapped with all the other native and depleted blood samples and is C-reactive protein (CRP), which is a well-established, yet unspecific, melanoma biomarker [50]. Moreover, the high number of upregulated proteins in plasma- and serum-derived EVs of melanoma patients highlighted the benefit of using EVs as complementary melanoma biomarkers. Among the upregulated candidates in plasma-derived EVs were melanoma markers [51], such as MCAM, TNC, and TGFBI, which were not regulated in depleted plasma samples (Supplementary Fig. S8b). To conclude, the proteome of plasma and serum-derived EVs were unique and revealed the potential to detect more differences between healthy controls and melanoma patients. Thus, upscaling SEC–DIA-MS to a larger melanoma cohort will facilitate biomarker discovery in melanoma.

### Plasma vs serum comparisons in native, depleted, and EV-derived blood compartments reveal specific enrichment of cellular components in depleted plasma and serum

To assess how differences in the blood collection method affect biomarker discovery, we compared the proteins in plasma vs serum for each of the blood compartments (native, depleted, and EVs). Generally, we observed a high overlap of proteins in all compartments ranging from 69.8% in native samples, to 68.4% and 71.2% in depleted and EV samples, respectively (Supplementary Fig. S9a). We also observe the expected reduction in concentration of clotting factors such as for example fibrinogen A (log_2_FC native: 8.43; depleted: 7.39; EVs: 6.86) or coagulation factor XIII A (log_2_FC native: 2.14, depleted: 2.51, EVs: N/A). To analyze the differences in more detail, we performed GO enrichment analysis. In both native and depleted plasma, as well as in plasma-derived EVs, the presence of proteins associated with Gene Ontology (GO) terms related to nuclear compartments was enriched. This observation implied that variations stemming from blood collection procedures can manifest similarly across various sample types (Supplementary Fig. S9b, c). Conversely, there was no significant enrichment observed in native and depleted serum samples, arguing against large global changes between the two blood collections in these sample types. In the case of serum-derived EVs, the enriched Gene Ontology (GO) terms were linked to endosomal and endoplasmic reticulum (ER) membranes (Supplementary Fig. S9d). We conclude that dissimilarities between plasma and serum were most pronounced in depleted blood.

### Membrane proteins are differentially enriched in plasma and serum EVs

**Fig. 4:**
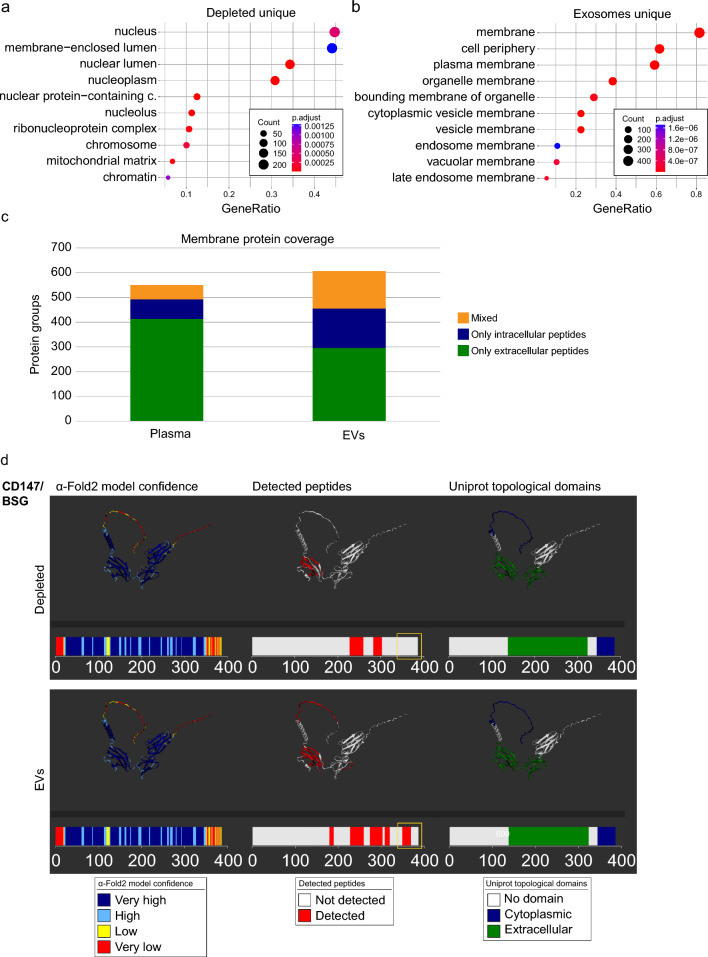
Membrane proteins are enriched in plasma and serum EVs. **a**, **b** GO terms of enrichment analysis of proteins identified in depleted (**a**) and EV-derived (**b**) blood biopsies. **c** Quantitation of membrane protein coverage in plasma and EV-derived plasma samples. Membrane proteins were identified peptides mapped exclusively to the intracellular or exclusively to the extracellular domains of the proteins are labelled in blue and green, respectively. Membrane proteins were both intracellular as well as extracellular peptides were identified are indicated in orange. **d** Protein structures of membrane protein Basigin (CD147/BSG) in depleted (top) and EV-derived (bottom) plasma biopsies are shown. The left panel shows the alpha-fold2 protein structure with associated model confidence. Very high confidence is indicated in dark blue (per-residue confidence score, pLDDT > 90), high confidence in light blue (90 > pLDDT > 70), low confidence in yellow (70 > pLDDT > 50), and very low confidence in red (pLDDT < 50). The middle panel shows the peptides that were identified by mass spectrometry as red lines on the size scale and as colouring on the 3D map. The right panel shows the corresponding UniProt topological domains of the protein. The yellow box highlights the cytosolic peptides seen exclusively in the EV samples

To study the different protein pools in native, depleted, and EV blood compartments in more detail, we examined which types of proteins are uniquely detected in these three compartments (Fig. 1d). In native blood samples, enrichment analysis yielded no significant enrichment (padj < 0.05) for the GO category “cellular component”. In depleted blood samples, mainly proteins related to nuclear compartments were enriched in the protein pool unique for this compartment (Fig. 4a). Proteins unique in EVs were significantly enriched for GO terms of cellular components associated with vesicles and membrane proteins (Fig. 4b), potentially pointing to an advantage of using EVs to isolate membrane associated biomarkers, especially those coming from the diseased tissue. Taking advantage of the peptide resolution that mass spectrometry provides, we took a closer look at the topological origin of the peptides that we quantified in depleted plasma and plasma-derived EVs (Fig. 4c). While, in depleted plasma, we primarily detected peptides mapping to the extracellular region of the membrane proteins (75.1% of membrane proteins), the EV samples contained a significant portion of peptides mapping to the intracellular domain or both intracellular and extracellular domain (mixed) domains of membrane proteins (51.1%). The reduction of intracellular peptides detected in depleted plasma was attributed to two factors: the identification of fewer proteins with exclusively intracellular peptides and the detection of a reduced number of intracellular peptides within proteins possessing both cytoplasmic and intracellular (mixed) domains (Fig. 4c). Taken together, identification of a large portion of peptides from intra- and extracellular domains (24.9%) in EV membrane proteins demonstrates that we were able to capture intact membrane proteins bound to EVs (Table 1). For example, Basigin (CD147/BSG) peptides detected in the depleted blood compartment mapped exclusively to the extracellular domain (Fig. 4d, Table 1), suggesting that cleaved Basigin is present in the blood. In contrast, comparable percentages of Basigin-specific peptides mapped to cytoplasmic (21.4%) and extracellular (78.6%) domains in plasma-derived EVs. Similarly, detected peptides of integrin beta1 (ITB1), the transferrin receptor (TFR1) and ADAM10 mapped to both, cytoplasmic and extracellular domains in plasma-derived EVs suggesting the presence of intact transmembrane proteins (Supplementary Fig. S10a–c, Table 1). Thus, parallel deep proteomic profiling of depleted plasma and plasma-derived EVs allowed us to address differences in soluble vs membrane bound proteins. Together with the observation that different protein types were enriched in depleted blood compared to EVs, this suggests that profiling both compartments will likely be complementary for melanoma biomarker discovery.

**Table 1:** Peptides detected by mass spectrometry in EV and depleted plasma compartments

		EVs			Plasma		EVs		Plasma		Reference	
UniProt ID	Gene Name	OP^a^	IP^b^	M	OP	IP	IP (%)^c^	OP (%)	IP(%)	OP (%)	IP(%)	OP (%)
P23470	PTPRG	2.00	13.00	NA	103.00	0.00	86.67	13.33	100.00	0.00	49.07	50.93
P02786	TFRC	96.00	12.00	NA	234.00	0.00	11.11	88.89	100.00	0.00	8.80	91.20
O14672	ADAM10	55.00	10.00	NA	25.00	0.00	15.38	84.62	100.00	0.00	7.05	92.95
Q9BX67	JAM3	19.00	10.00	NA	4.00	0.00	34.48	65.52	100.00	0.00	18.15	81.85
P27824	CANX	20.00	9.00	NA	95.00	0.00	31.03	68.97	100.00	0.00	16.49	83.51
P05107	ITGB2	69.00	9.00	NA	91.00	0.00	11.54	88.46	100.00	0.00	6.34	93.66
P05556	ITGB1	97.00	8.00	NA	177.00	0.00	7.62	92.38	100.00	0.00	5.85	94.15
Q9Y624	F11R	38.00	7.00	NA	22.00	0.00	15.56	84.44	100.00	0.00	16.33	83.67
P30203	CD6	12.00	7.00	NA	4.00	0.00	36.84	63.16	100.00	0.00	38.79	61.21
O60486	PLXNC1	5.00	6.00	NA	6.00	0.00	54.55	45.45	100.00	0.00	39.83	60.17
P35613	BSG	22.00	6.00	NA	4.00	0.00	21.43	78.57	100.00	0.00	11.92	88.08
P08648	ITGA5	31.00	5.00	NA	75.00	0.00	13.89	86.11	100.00	0.00	2.94	97.06
Q02763	TEK	8.00	5.00	NA	61.00	0.00	38.46	61.54	100.00	0.00	32.93	67.07
P48960	ADGRE5	4.00	5.00	NA	35.00	0.00	55.56	44.44	100.00	0.00	15.37	84.63
Q13444	ADAM15	11.00	5.00	NA	12.00	0.00	31.25	68.75	100.00	0.00	17.87	82.13
O43157	PLXNB1	13.00	4.00	NA	141.00	0.00	23.53	76.47	100.00	0.00	29.85	70.15
Q13308	PTK7	7.00	4.00	NA	89.00	0.00	36.36	63.64	100.00	0.00	34.18	65.82
P13612	ITGA4	36.00	4.00	NA	23.00	0.00	10.00	90.00	100.00	0.00	3.38	96.62
P01911	HLA-DRB1	18.00	4.00	NA	10.00	0.00	18.18	81.82	100.00	0.00	8.33	91.67
P23229	ITGA6	90.00	3.00	NA	84.00	0.00	3.23	96.77	100.00	0.00	4.97	95.03
O95866	MPIG6B	9.00	3.00	5	6.00	0.00	17.65	52.94	100.00	0.00	38.42	61.58
P15529	CD46	21.00	3.00	NA	5.00	0.00	12.50	87.50	100.00	0.00	7.72	92.28

^a^OP, Outer peptides list the number of identified peptides mapping to the extracellular domain of the transmembrane protein

^b^IP, Inner peptides list the number of identified peptides mapping to the intracellular domain of the transmembrane protein

^c^The percentage in column 8–13 refers to the sum of detected inner and outer peptides of the corresponding protein (column 1, 2) in the given blood compartment or the reference. The topology classification of UniProt was used as reference. Only proteins with > 3 peptides detected are shown

### Plasma and serum-derived EV proteins are functionally distinct compared to non-vesicular plasma and serum proteins

**Fig. 5:**
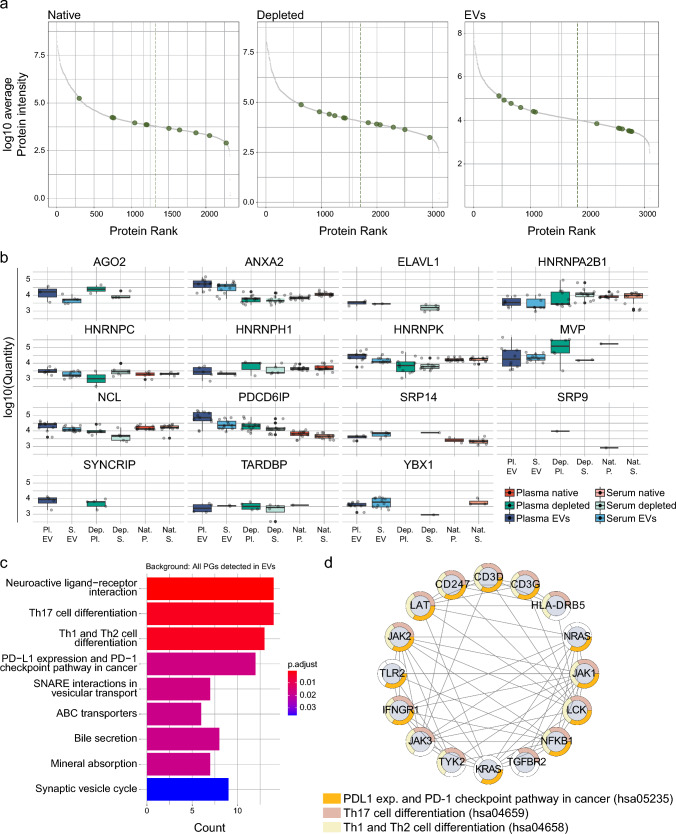
Plasma- and serum-derived EV proteins are functionally distinct compared to non-vesicular plasma and serum proteins. **a** Average protein intensity plotted vs ranked protein abundance with proteins highlighted belonging to RNA-binding proteins previously identified in EVs. **b** Quantification of detected proteins in A across native plasma (dark red), native serum (light red), depleted plasma (dark green), depleted serum (light green), plasma-derived EVs (dark blue), and serum-derived EVs (light blue). Black lines represent the median and the upper and lower limit of the box depict the 25 and the 75 percentile, respectively. **c** KEGG pathway enrichment analysis of proteins uniquely presents in EVs against all identified proteins. Note that several pathways related to T-cell biology are significantly enriched in EVs compared to depleted and native blood. **d** PPI network created using the string database (https://string-db.org/) and the enriched proteins found exclusively in EVs. Colored segments label proteins enriched for the indicated KEGG/Reactome terms: dark yellow segments correspond to the term “PDL1 expression and PD-1 checkpoint pathway in cancer”, light yellow segments to the terms “Th1 and Th2 cell differentiation”, and beige segments to the term “Th17 cell differentiation”

Given the observed difference in protein type and structure between EVs and plasma, we next assessed whether EV proteins whether EV-associated proteins exhibited distinct functional characteristics compared to non-vesicular proteins. EVs play a crucial role in cell communication in part mediated by transfer of RNAs including mRNAs, miRNAs, and long non-coding RNAs (lncRNAs) to recipient cells [15, 52, 53]. To transport of these RNAs to EVs, the RNAs form complexes with RNA binding proteins (RBPs). We therefore investigated whether the proteins exclusively found in EVs belong to the category of potential RBPs. Remarkably, a substantial portion of these unique EV proteins, specifically 209 out of 489, have been characterized as potential RBPs (source: https://r-deep.dkfz.de/). Furthermore, we quantified the abundance of RBPs that have been reported to be associated with EVs in the literature [54]. In our analysis, we identified 14 of the 23 reported EV-associated RNA-binding proteins in plasma-derived EVs and 13 in serum-derived EVs (Fig. 5a, b). Among these identified RBPs ANXA2, Alix (PDCD6IP) and YBX1 were specifically enriched in EV samples compared to unenriched plasma or serum (Fig. 5a, Supplementary Fig. S11a, b), consistent with their role in miRNA loading [55–57]. Given the involvement of YBX1 in (oncogenic) translation and its reported role in tRNA sorting into EVs [58–61], we further explored the presence of amino-acyl tRNA synthetases, which are the enzymes responsible for loading amino acids onto tRNAs [62, 63]. We observed a slight enrichment of aminoacyl-tRNA synthetases (ARS) in EVs when compared to native samples, although no such enrichment was observed in comparison to depleted samples [62, 63] (Supplementary Fig. S12a). While mitochondrial ARS were missing, we detected most of the exclusively cytosolic ARS in EVs (Supplementary Fig. S12b). Significantly, 13 of the 18 ARS identified in EVs were exclusive to melanoma patients, while the remaining 5 ARS showed increased abundance in melanoma patient-derived EVs compared to healthy controls. This contrasted with depleted plasma, where 83.3% of ARS were common to both healthy controls and melanoma patients. This observation hinted at a potential role of EV-mediated ARS sequestration in melanoma.

To further explore functional aspects of EV proteins, we conducted a pathway enrichment analysis focused on proteins exclusively identified in EVs. Our analysis revealed several enriched pathways including vesicle-related pathways such as “SNARE interactions in vesicular transport” and “Synaptic vesicle cycle”. Moreover, we found enrichment of pathways closely tied to T-cell biology within this unique EV proteome, notably including "PDL1 expression and PD-1 checkpoint pathway in cancer," "Th17 cell differentiation," and "Th1 and Th2 cell differentiation" (Fig. 5c). Visualisation of these enriched KEGG terms in a protein interaction network revealed EV-specific proteins associated with the T-cell receptor (e.g., CD3D, CD3G, and CD247), and proteins linked to the immunological synapse (e.g., LCK and LAT), as EV-specific. Taken together, analysis of the plasma and serum EV proteome revealed structurally, and functionally distinct proteins compared to unenriched plasma/serum proteomes and an enrichment of proteins related to T-cell signalling, again showing potential for the combined profiling of both compartments.

## Discussion

Here we present SEC–DIA-MS, a workflow for deep protein profiling of plasma- and serum-derived EVs and use of a high-abundant protein depletion method to compare EV proteins to plasma- and serum-derived samples. SEC–DIA-MS allowed for improved identification and quantification of plasma-derived EV proteins compared to previous melanoma proteomic studies of plasma derived EVs [36–39]. It is worth noting though that differences in experimental parameters have limited the direct comparability between our study and the previous investigations. For instance, in the study by Pietrowska et al., EVs were subjected to further immunoselection using anti-CSPG4 antibodies. Although reported protein group identifications combine both CSPG4 positive and negative samples, further enrichment may have led to potential sample loss. Another source of variation was the sample input used, which was 5 × higher in the mentioned study and not explicitly specified in the other studies. In our work, we analyzed 200 µL of plasma or serum from a cohort consisting of three healthy donors and six melanoma patients, quantifying 2896 or 2794 exosome-associated proteins, where the majority of protein identifications were contributed by the melanoma samples. Concentrations and lysis of EVs after SEC combined with the use of the most advanced proteomic equipment were likely the dominant contributors to improvements.

Enrichment of the GO term “extracellular vesicle”, the presence of several exosome markers, including tetraspanins CD9, CD63, CD81, PDCD6IP, TSG101, and syntenin-1 and a high overlap (94/100) with the top 100 exosome proteins listed in Exocarta confirmed enrichment of exosomes in our EV preparations. This aligns with recent findings where SEC of platelet-poor plasma outperformed other EV isolation methods in the detection of EV markers [64]. Interestingly, we observed that among the commonly recognized EV markers, CD81 exhibited higher abundance in plasma compared to serum-derived EVs in our study. However, this was unlikely attributed to platelet-derived EVs in serum, as previous studies did not detect CD81 on platelets [65, 66]. Moreover, the effective application of SEC combined with DIA-MS has been employed in the analysis of serum-derived extracellular vesicles (EVs) within a non-human cancer model. In this study, researchers successfully identified cathelicidin-3 (CATH3), an antimicrobial peptide, as a potential biomarker for early cancer diagnosis in the Tasmanian devil [67, 68].

Besides the high recovery of protein identifications, analysis with three technical replicates per sample type showed high reproducibility of the workflow. Additional experimental steps such as EV isolation and concentration as well as the volume normalized injection into the LC–MS/MS increased the CV of technical EV replicates; whereas EVs also displayed higher biological variance than plasma and native samples. Generally, we observed minor differences between plasma and serum EVs in terms of particle size and numbers and protein group identifications showed a high overlap of 71.2%. These findings were in line with a recent study that performed a pairwise comparison of plasma- and serum-derived EVs where a large overlap (87.4%) of plasma and serum EV proteins was detected [69]. However, these comparisons should be interpreted with caution as they are influenced by sample quality and the anticoagulant used during blood collection [70–73]. For example, large amounts of platelet activation in serum samples can drastically change EV composition in the two sample types and therefore lead to a high abundance of platelet EV proteins [74, 75]. Thus, samples processed without two-step centrifugation or with other anti-coagulants than EDTA may vary in their EV proteome profile.

A major challenge for proteomic studies from blood liquid biopsies is the high abundance of certain blood proteins contributing to the high dynamic range of human blood, which is estimated to be in the range of 12–13 orders of magnitude [76]. Analysis of high-abundance proteins that were targeted with antibodies in the depleted blood type samples confirmed efficient depletion. This led to strongly increased proteomic sensitivity, underscored by the detection of the melanoma biomarker CRP in differential abundance analysis of plasma and serum samples of only nine human subjects. Showcasing the effective EV isolation, EV samples showed fewer high-abundant proteins compared to native blood samples. The least reduction compared to native plasma was seen for IGHM, A2M, APOA2, and FGA, which have been detected in the protein corona of EVs [40]. In contrast, the levels of ALB, TF, TTR, and ORM1 were most significantly reduced in comparison to native plasma. Recent studies have demonstrated that combining size-exclusion chromatography (SEC) with density cushion can effectively reduce lipoprotein contaminants in plasma-derived EV samples that may co-isolate with EVs [77]. Additionally, magnetic bead-based EV isolation methods, such as MagCapture, have shown promising results in reducing protein contaminants in cell culture supernatants, nearly matching the performance of size-exclusion chromatography [78]. However, it is important to note that MagCapture is not compatible with EDTA-containing samples, requiring the exploration of alternative magnetic bead-based approaches for plasma samples. Moreover, even though protein contaminants can be removed depending on the choice of EV isolation method, the removal of the protein corona remains a barrier to comprehensive proteome profiling. On the other hand, in an interesting approach, the concept of corona formation by nanoparticles introduced into biofluids has been leveraged to isolate EVs by fine-tuning these protein nano-interactions to profile the blood proteome. This technique has not yet been applied to EVs’ profiling though. [79, 80]. Therefore, techniques such as SEC–DIA-MS, which allow for increased depth of proteomic profiling, are required to overcome these shortcomings.

The improved detection of EV proteins with SEC–DIA-MS and the efficient depletion of high abundant proteins in unenriched blood samples allowed us to compare the EV proteome with the proteome of non-vesicular plasma and serum liquid biopsies. Notably, PCA analysis and unsupervised hierarchical clustering revealed the presence of different protein populations in native, depleted, and EV-derived plasma and serum compartments. This suggested that our SEC–DIA-MS approach will likely be complementary to deep-profiling of depleted plasma and serum in terms of biomarker discovery and disease detection. Analogously, differences between EVs and plasma compartments were recently reported for miRNAs [81]. In particular, plasma EVs were not only enriched in membrane-bound protein markers, but thorough integration of mass spectrometry analysis with 3D modeling showed that the plasma compartment of most membrane bound proteins was present as free-floating, cleaved, extracellular domains; whereas, in EVs, these membrane proteins were complete. Several membrane proteins as, for example, Basigin, ITB1, TFR1, ADA10, and NOTC4 were predicted to be truncated in plasma but whole in plasma EVs. This is consistent with reports about proteolytic cleavage of most of those proteins [82–86]. Basigin, in particular, has emerged as a promising candidate for therapeutic intervention in melanoma due to its multifaceted role in melanoma progression, influencing cell viability, apoptosis, proliferation, and invasion. Notably, one of the functions of Basigin, such as cleavage of MMP2 released by fibroblasts, has been specifically attributed to its cleaved form. This underlines the importance of distinguishing structural differences of protein biomarkers and might show that EVs are enriched with membrane proteins coming from the diseased organ or tumor, in this case.

Several RNA-binding proteins have been reported to target small RNAs and miRNAs to EVs. Consistently, we detected previously published RNA-binding proteins [54] in both plasma- and serum-derived EV samples. On the other hand, we did not detect hnRNPG, hnRNPH1, HuR, IGF2BP1, MEX3C, FUS, TDP-43, LIN28, and QKI, which have been reported to be present in EVs. The absence could be due to biological differences in EVs or limited sensitivity. RBPs that were specific to EVs included YBX1, a known prognostic marker for several cancers [87–89], which has been linked to melanoma progression by regulating MIA-dependent expression [90]. We also observed enrichment of the KEGG term “SNARE interactions in vesicular transport” including several syntaxins, which have recently been shown to be more abundant in smaller rather than larger EVs [91]. Notably, we also detected enrichment of proteins related to T-cell biology including T-cell receptor components and downstream signalling proteins of the JAK–STAT pathway. T-cell exosomes have been described to have antitumoral effects. For example, T-cell-derived exosomal PD1 was shown to interact with PD-L1 on breast cancer cells and trigger clathrin-mediated endocytosis, allowing the immune system to recognize cancer cells [92] and T-cell-derived exosomal FASL expression was associated tumor metastasis [93]. Taken together, EV isolation did not only improve protein group identifications compared to native plasma, but also resulted in an enrichment of specific sets of proteins. It is worth noting that a recent study demonstrated a substantial enhancement in protein identifications, on a scale similar to our investigation, simply by fractionating plasma through centrifugation at 20,000 g for 30 min. Nonetheless, the extent to which proteins from larger vesicles and potential organelle fragments contributed to the substantial increase in the number of identified proteins remained unaddressed in that study.

Comparison of age- and gender-matched healthy control and melanoma patients revealed several unique proteins especially in melanoma EVs. This is in line with the reports of EV generation by melanoma [94–96], which would lead to a different EV population in plasma, finally leading to these unique identifications. For example, several ARS (AARS1, CARS1, DTD1, EARS, EPRS1, FARSB, GARS1, HARS1, LARS1, NARS, RARS1, SARS1, and VARS1) could only be detected in melanoma EVs, whereas WARS and YARS were more abundant in EVs from melanoma patients compared to EVs of healthy donors. These findings align with recent studies highlighting the emerging roles of ARS as important regulators and potential biomarkers in various cancer types [96]. Beyond their function in translation control, ARS have emerged as key players in maintaining cellular homeostasis. [97]. Beyond controlling translation, they have been recognized as regulators of cellular homeostasis. For example, increased cellular WARS levels adapt cancer cells to nutritional stress in response to tryptophan degradation [98]. Whether EVs contribute to ARS-mediated homeostasis remains to be explored. Moreover, proteins related to melanoma biology (e.g., MET, CSPG4/MCSP) were specifically upregulated in melanoma compared to healthy EVs, but showed no differential abundance in depleted plasma, showing the sensitivity and specificity of the EV preparation. MET has previously been identified as a biomarker for melanoma [31]. Similarly, CSPG4/MCSP has been described as a marker for circulating melanoma cells and EV marker for malignant melanoma [99, 100]. In conclusion, SEC–DIA-MS revealed differences between EV and non-vesicular proteome of melanoma blood biopsies and specific presence/upregulation of proteins exclusively in melanoma EVs, thus indicating the complementarity of the two matrices for biomarker discovery, including biomarker discovery for early detection (stage I) and cancer staging (stage I-IV). Therefore, we expect SEC–DIA-MS to be a useful tool for further large-scale melanoma studies. Moreover, given the importance of EVs in physiology and different diseases [101], SEC–DIA-MS can also be applied to other biomarker studies.

### Supplementary Information

Below is the link to the electronic supplementary material.

Supplementary file1 (DOCX 33634 KB)

## Data Availability

All of the mass spectrometry data can be found on MassIVE (https://massive.ucsd.edu/).
