# Supplementary material for: Size-exclusion chromatography combined with DIA-MS enables deep proteome profiling of extracellular vesicles from melanoma plasma and serum
Source: Cell Mol Life Sci. 2024 Feb 14;81(1):90. doi: 10.1007/s00018-024-05137-y (PMC10867102; doi:10.1007/s00018-024-05137-y)
Supplement: Supplementary file 1 — Supplementary file1 (DOCX 33634 KB) [file 18_2024_5137_MOESM1_ESM.docx]

**Size-exclusion chromatography combined with DIA-MS enables deep proteome profiling of extracellular vesicles from melanoma plasma and serum**

Evelyn Lattmann^1^, Luca Räss^2^, Marco Tognetti^2^, Julia M. Martínez Gómez^1^, Valérie Lapaire^1^, Roland Bruderer^2^, Lukas Reiter^2^, Yuehan Feng^2^, Lars M. Steinmetz^3,4,5*^, Mitchell P. Levesque^1*^

* Correspondence:

Lars.Steinmetz@stanford.edu

Mitchell.Levesque@usz.ch

^1^ Department of Dermatology, University Hospital Zurich, University of Zurich, Schlieren, Switzerland

^2^ Biognosys AG, Schlieren, Switzerland

^3^ Stanford Genome Technology Center, Stanford University, Palo Alto, CA, USA

^4^ Department of Genetics, Stanford University School of Medicine, Stanford, CA, USA

^5^ Genome Biology Unit, European Molecular Biology Laboratory, Heidelberg, Germany

**Supplementary Fig. S1 Mass spectrometry response of all analyzed samples (**a) The box plots represent the mass spectrometry response for each analyzed plasma sample (patient samples and pooled samples) comprising plasma-derived EV-samples (dark blue), matching depleted plasma samples (dark green), and matching native plasma samples (dark red), respectively. (b) The box plot represents the mass spectrometry response for each analyzed serumsample (patient sample and pooled sample) comprising serum-derived EV-samples (light blue), matching depleted serum samples (light green), and matching native plasma samples (light red), respectively. Black lines represent the median and the upper and lower limit of the box depicts the 25 and the 75 percentile, respectively. Orange diamonds indicate the mean value.

**Supplementary Fig. S2 EV characterization (**a) Size profiles of all isolated EV samples acquired with nano flow cytometer (excluding quality control samples). (b) Absorption measurements at 280 nm for all analyzed EV samples (excluding quality control samples). Data points derived from healthy controls are labelled in white, from melanoma stage III patients in light brown and from melanoma stage IV patients in dark brown (c) Scatter plot illustrating the relationship between protein group identifications and particle concentration of plasma-derived EV samples (left) and serum-derived EV samples (right). Each data point represents an individual subject. The solid line across the plot represents the optimal-fit linear regression line with the corresponding coefficient of determination R-squared (R²) value given next to it.

**Supplementary Fig. S3 Enrichment of exosome markers in plasma- and serum-derived EVs.** (a) Each graph represents the protein intensity of detected proteins plotted vs protein abundance for a blood (plasma, left; serum; right) compartment (native, top; depleted, middle; EVs, bottom) from one patient. Red dots indicate classical exosome markers (CD151, CD63, CD81, CD9, FLOT1, PDC6I, SDCBP, TSG101). (b) Protein sequence coverage and (c) number of detected peptides of exosome markers detected in native, depleted and EV-derived plasma and serum samples. Black lines represent the median and the upper and lower limit of the box depicts the 25 and the 75 percentile, respectively

**Supplementary Fig. S4 EV Organelle markers are absent or reduced in EV samples** (a) Organelle markers absent in EV isolations. (b) Organelle markers diminished in EV isolations. Log-transformed protein quantity, the number of individual peptides and protein percentage coverage of organelle markers in native, depleted and EV-derived plasma and serum samples, respectively are plotted. Black lines represent the median and the upper and lower limit of the box depicts the 25 and the 75 percentile, respectively

**Supplementary Fig. S5 Reproducibility analysis of the workflow**

(a) Measurement of protein absorption at 280 nm of three technical (n=3) replicates of plasma and serum samples, respectively. (b) Particle counts of isolated exosomes of three technical replicates (n=3) of plasma and serum samples, respectively. (c) Mean size of particles measured in B. (d) Corresponding size distribution of particles measured in B. (e) Coefficient of variation (CV) of protein group quantities derived from sample processing (“technical”, n = 3) and across all analyzed patient samples (“biological”, n = 9). (f) UpSet plot showing the number of shared proteins measured in three technical replicates of plasma (left, n=3) and serum (right, n=3) in native (top), depleted (middle) and exosome (bottom) samples. The horizontal bars show the number of proteins identified per sample while the vertical bars display the number of overlapping proteins per subsets which are visualized by the dotted lines. (g) Protein groups, modified peptides, peptides and precursors identified in native, depleted and EV-derived plasma and serum, respectively. For each compartment three technical replicates (n=3) were assessed

**Supplementary Fig. S6 Efficient depletion of highly abundant blood proteins and enrichment of RNA binding proteins in EV samples** (a-h) Depletion (a), platelet (c), erythrocyte (e) and coagulation (g) score of native, depleted and EV-derived blood samples. The score is calculated based on the corresponding proteins plotted in B, D, F and H. Quantification of proteins that were targeted by antibodies for plasma depletion(b) and a selected protein panel to assess sample quality (d, f, h). All quantities are given for native (red), depleted (green) and EV (blue) compartments. Black lines represent the median and the upper and lower limit of the box represent the 25 and the 75 percentile, respectively. (i) Proteins ranked based on abundance versus protein average intensity, represented by the dotted vertical line. Dashed lines represent the average rank of the depicted categories

**Supplementary Fig. S7 Protein identification reproducibility in native and depleted blood** UpSet plot showing the number of shared proteins among all analyzed samples in native (a) and depleted (b) plasma and serum, respectively. The horizontal bars (red and green) show the number of proteins identified per sample while the vertical bars (black) display the number of overlapping proteins per subsets which are visualized by the dotted lines

**Supplementary Fig. S8 Deep protein profiling reveals potential of plasma and serum EVs for melanoma protein biomarker discovery** (a) Log-fold change of upregulated proteins in melanoma patient samples compared to healthy control samples. Log2FC were assessed for native (red), depleted (green), and EV-derived (blue) plasma and serum compartments. A cut-off of LogFC > 2 or < -2 and p-value < 0.05 was applied. Note that several proteins are differentially upregulated in EVs derived from melanoma patients compared to EVs derived from healthy donors. Black lines represent the median and the upper and lower limit of the box depicts the 25 and the 75 percentile, respectively. (b) Quantification of melanoma markers^44^ in plasma-derived EVs and plasma of melanoma patients and healthy donors. Black lines represent the median and the upper and lower limit of the box depicts the 25 and the 75 percentile, respectively

**Supplementary Fig. S9 Plasma vs serum comparisons in native, depleted and EV-derived blood** (a) Venn diagram displaying the overlap of plasma and serum protein identification among native (left), depleted (middle) and EV-derived (right) blood biopsies. (b-d) GO term for enrichment analysis of plasma vs serum protein identifications in native plasma (b), depleted plasma (c), plasma-derived EVs and serum-derived EVs (d)

**Supplementary Fig. S10 Plasma and EV membrane proteins differ in their protein structure** (a-c) Protein structures of membrane proteins ITB1 (a), TFR1 (b), ADAM10 (c) in depleted (top) and EV-derived (bottom) blood biopsies are shown. The left panel shows the alpha-fold2 protein structure with associated model confidence. Very high confidence is indicated in dark blue (per-residue confidence score, pLDDT>90), high confidence in light blue (90>pLDDT>70), low confidence in yellow (70>pLDDT>50) and very low confidence in red (pLDDT<50). The middle panel shows the peptides that were identified by mass spectrometry as red lines on the size scale and as colouring on the 3D map. The right panel shows the corresponding UniProt topological domains of the corresponding proteins. The yellow box highlights the cytosolic peptides seen exclusively in the EV samples

**Supplementary Fig. S11 RNA binding proteins are enriched in EV-derived samples** (a, b) Each graph represents the protein sequence coverage (a) and the number of detected peptides (b) of EV RNA binding proteins detected in native, depleted and EV-enriched blood samples. Black lines represent the median and the upper and lower limit of the box depicts the 25 and the 75 percentile, respectively

**Supplementary Fig. S12 Blood-derived EVs are enriched in ribosome proteins and contain all cytosolic aminoacyl-tRNA synthetases (except CARS, IARS and LARS)** (a) Average protein intensity plotted vs protein rank of proteins mapping to the KEGG annotation “Aminoacyl-tRNA biosynthesis” in native, depleted and EV-derived blood compartments. (b) Quantification of ARS in depleted plasma and plasma-derived EVs of melanoma patients and healthy donors. Black lines represent the median and the upper and lower limit of the box depicts the 25 and the 75 percentile, respectively.
